# Parent's perception on end-of-life care in Brazil

**DOI:** 10.1186/cc12470

**Published:** 2013-03-19

**Authors:** P Lago, J Piva, G Halal, M Halal

**Affiliations:** 1 Hospital de Clinicas de Porto Alegre, Brazil

## Introduction

The aim of the study is evaluate the perceptions of parents of children who died in two Brazilian pediatric ICUs.

## Methods

An exploratory-descriptive study with a qualitative approach in the PICU of Hospital São Lucas and Hospital de Clinicas de Porto Alegre involving 15 parents of children who died. Data collection was performed through three steps: (a) the researchers contacted the parents through a telephone call to invite them to attend the hospitals; (b) at the hospital, the doctors who assisted the children clarified doubts about the therapy offered; and (c) an interview was carried out by two researchers not involved in the care. Data analysis was performed using the technique of thematic content analysis.

## Results and conclusion

The research shows that the difficulty of communication is a factor that impacts negatively on the grieving process. Moreover, it stresses the importance for parents to rediscuss the moment of their child's death with health professionals.
